# Mean Platelet Volume (MPV): New Perspectives for an Old Marker in the Course and Prognosis of Inflammatory Conditions

**DOI:** 10.1155/2019/9213074

**Published:** 2019-04-17

**Authors:** Aleksandra Korniluk, Olga Martyna Koper-Lenkiewicz, Joanna Kamińska, Halina Kemona, Violetta Dymicka-Piekarska

**Affiliations:** Department of Clinical Laboratory Diagnostics, Medical University of Bialystok, ul. Waszyngtona 15A, 15-276 Białystok, Poland

## Abstract

Platelet size has been demonstrated to reflect platelet activity and seems to be a useful predictive and prognostic biomarker of cardiovascular events. It is associated with a variety of prothrombotic and proinflammatory diseases. The aim is a review of literature reports concerning changes in the mean platelet volume (MPV) and its possible role as a biomarker in inflammatory processes and neoplastic diseases. PubMed database was searched for sources using the following keywords: platelet activation, platelet count, mean platelet volume and: inflammation, cancer/tumor, cardiovascular diseases, myocardial infarction, diabetes, lupus disease, rheumatoid arthritis, tuberculosis, ulcerative colitis, renal disease, pulmonary disease, influencing factors, age, gender, genetic factors, oral contraceptives, smoking, lifestyle, methods, standardization, and hematological analyzer. Preference was given to the sources which were published within the past 20 years. Increased MPV was observed in cardiovascular diseases, cerebral stroke, respiratory diseases, chronic renal failure, intestine diseases, rheumatoid diseases, diabetes, and various cancers. Decreased MPV was noted in tuberculosis during disease exacerbation, ulcerative colitis, SLE in adult, and different neoplastic diseases. The study of MPV can provide important information on the course and prognosis in many inflammatory conditions. Therefore, from the clinical point of view, it would be interesting to establish an MPV cut-off value indicating the intensity of inflammatory process, presence of the disease, increased risk of disease development, increased risk of thrombotic complications, increased risk of death, and patient's response on applied treatment. Nevertheless, this aspect of MPV evaluation allowing its use in clinical practice is limited and requires further studies.

## 1. Introduction

Thrombocytes are the smallest and yet extremely reactive blood morphotic components. They are involved first of all in the processes of fibrosis and maintenance of normal hemostasis. Recent studies have provided abundant evidence for their multifunctional nature. Blood platelets are the first to accumulate at the site of damage, where they change in shape and show formation of pseudopodia, local release of cytoplasmic granular content, and aggregation, when activated by classical agonists, such as ADP, TXA2, PAF, and inflammatory cytokines, e.g., IL-1, IL-6, and TNF alpha [1]. Thus, they initiate fibrosis and inflammatory processes.

Literature data indicate that mean platelet volume (MPV) can provide important information on the course and prognosis in many pathological conditions, such as cardiovascular diseases, respiratory diseases, Crohn's disease, rheumatoid arthritis, juvenile systemic lupus erythematous, diabetes mellitus, and the majority of neoplastic diseases (Tables 1 and 2) [2–36]. Therefore, the aim of the current paper is a review of literature reports concerning changes in MPV and its possible role as a biomarker in various inflammatory processes and neoplastic diseases.

### 1.1. Search Strategy

We searched PubMed database was searched for sources using the following keywords: platelet activation, platelet count, mean platelet volume and: inflammation, cancer/tumor, cardiovascular diseases, myocardial infarction, diabetes, lupus disease, rheumatoid arthritis, tuberculosis, ulcerative colitis, renal disease, pulmonary disease, influencing factors, age, gender, genetic factors, oral contraceptives, smoking, lifestyle, methods, standardization, and hematological analyzer. Preference was given to the sources which were published within the past 20 years.

## 2. Blood Platelet Formation

Blood platelets are formed during thrombocytopoiesis as nonnucleated and discoidal fragments of megakaryocyte cytoplasm. Thrombopoietin (Tpo) is a hematopoietic cytokine playing a major role in the proliferation and differentiation of megakaryocytes and in consequence in blood platelet formation [37]. The activation of c-MPL receptor for Tpo on the surface of a megakaryocyte leads to cell proliferation, intracellular synthesis of platelet proteins, and loss of proliferating capacity. Next, the polyploidal nucleus of megakaryocyte is formed due to repeated processes of endomitosis [37], leading to enhanced cell metabolism, formation of the system of membranes, cell organelles, and specific granules, which is necessary for normal platelet formation [38]. The number and size of the originating platelets depend on the ploidy degree of megakaryocytes. The greater the ploidy of the megakaryocyte nucleus, the more cytoplasm and specific platelet structures it has. When activated, the cell forms cytoplasmic processes similar to pseudopodia, called proplatelets, which constitute an indirect form between megakaryocyte and thrombocyte [39]. Proplatelets are long cytoplasmic processes of megakaryocytes containing organelle characteristic of thrombocytes but without distinct border zones. Mature proplatelets are released to bone marrow vessels and being smaller than stem cells they are able to migrate to peripheral vessels. Proplatelets present in, e.g., pulmonary vessels can be even 100 times larger than blood platelets, thus indicating that their cytoplasm undergoes fragmentation already in the peripheral blood, giving rise to thrombocytes [40].

The activation of megakaryocytes and enhanced release of thrombocytes are mainly stimulated by thrombopoietin. It has been demonstrated that in inflammatory conditions, also IL-6, IL-1, and TNF-*α* can stimulate precursor cells of blood platelets [41]. The action of IL-6 is associated with enhanced Tpo generation in the liver and its direct effect on megakaryocytes through the membranous receptor IL-6R. This means that blood platelet count may increase markedly in an inflammatory condition.

The provision of the adequate platelet count indispensable for the maintenance of hemostasis in physiological and pathological conditions is associated with generation and release of these cells from the bone marrow [41]. Inactivated thrombocytes, present in circulation, do not virtually alter their morphological parameters. Any deviations in the count, total platelet mass, morphology, and function depend on the factors that directly affect the ploidy of megakaryocytes, the maturity of progenitor cells, and the activation and wear of blood platelets during coagulation and inflammatory processes [42].

Thrombocytes are present in the blood for 8 to 12 days [43]. On average, the plasma of healthy individuals shows the platelet count of approximately 140,000-350,000/*μ*l, forming the circulating pool (around 70% of total platelet mass) and the so-called exchange pool (30% of thrombocytes), present in the spleen [44].

### 2.1. Blood Platelets and Inflammatory Response

The involvement of blood platelets in an inflammatory response is associated with the release of cytokines and chemokines that attract leukocytes and facilitate adhesion to endothelium at the site of damage. During the inflammatory process, blood platelets may interact with leukocytes by forming platelet-leukocyte aggregates [45]. These bindings are possible through adhesion proteins expressed on the cell surface during activation. Moreover, platelets support leukocytes to combat bacterial infections via direct contact, encapsulation of bacteria, and release of reactive oxygen species and platelet microbicidal proteins (PMP). Platelet growth factors, such as TGF-beta, PDGF, or VEGF, are also engaged in wound healing [46].

Recent research has shown the involvement of blood platelets in the development of neoplastic disease. It is suggested that interactions of cancer cells with thrombocytes allow their migration from the primary tumor and formation of metastases. Encapsulation of transformed cells by blood platelets protects them from recognition by the host immune system and enables their binding with adhesion proteins on endothelial surface [47]. The involvement of blood platelets in these processes is associated with changes in their count and morphology. During coagulation, the count may decrease due to platelet wear, whereas the activation of megakaryocytes by proinflammatory cytokines may lead to a considerable increase in the production and release of thrombocytes. In some diseases, specific alterations are noted in platelet parameters, which can be thus used as diagnostic markers of these conditions.

### 2.2. Platelet Morphological Parameters

Basic platelet parameters are assessed during a routine blood morphology test providing valuable information on blood platelet count (PLT), mean platelet volume (MPV), platelet distribution width (PDW), and plateletcrit (PCT). Modern hematological analyzers enable the assessment of the percentage of large platelets with MPV > 15 fl (P-LCR), the number of giant platelets with MPV > 20 fl (LP), the number of reticulated platelets (PLRET), microplatelets (PDMP), and the mean platelet component (MPC). The latest research has shown that the platelet parameters can contribute to the diagnosis of patient's general condition and have a prognostic value in some pathologies [48]. Although the routine assessment of the platelet parameters has been available for many years now, their clinical significance has not been fully elucidated, and their diagnostic usage has been limited. Nowadays, determination of PLT and MPV is commonly accepted and recommended by the International Committee for Standardization in Hematology (ICSH).

### 2.3. The Mean Platelet Volume (MPV)

The mean platelet volume (MPV) is a precise measurement of their dimension, calculated by hematological analyzers on the basis of volume distribution during routine blood morphology test. MPV ranges between 7.5 and 12.0 fl, whereas the percentage of large platelets should amount to 0.2-5.0% of the whole platelet population [49]. In physiological conditions, MPV is inversely proportional to the platelet count, which is associated with hemostasis maintenance and preservation of constant platelet mass [50]. This means that the increased production of platelets is accompanied by a reduction in their mean volume. In various pathologies, this physiological proportion is disturbed. Markedly enhanced or abnormal thrombocytopoiesis, increased wear, or the effect of activating factors on blood platelets may lead to changes in the proportions between MPV and PLT [51]. Therefore, possible application of these parameters to the diagnosis of certain diseases has been suggested. Moreover, MPV correlates with platelet activity and is thus considered a marker of platelet activity [52]. Blood platelets are not a homogenous population. Those with increased MPV (>15 fl) are often younger and characterized by higher reactivity than those with normal MPV. Their generation is associated with marked activation of megakaryocytes by cytokines, which increases the ploidy of these cells and enhances the release of larger platelets [53]. It is also suggested that large thrombocytes show a greater content of cell granules, display higher expression of adhesion molecules, and undergo faster activation, which results in platelet hyperactivity and increased risk of clot formation [54]. Elevated MPV correlates with increased platelet aggregation, enhanced synthesis, and release of thromboxane TXA2 and *β*-thromboglobulin [55].

## 3. MPV in Inflammation

In healthy individuals, the increased platelet count, via feedback, leads to considerable inhibition of Tpo synthesis by the liver and in consequence causes platelet release by megakaryocytes, which is to maintain constant platelet mass. However, in patients with ongoing inflammation, the increasing concentration of proinflammatory cytokines, mainly IL-6, can lead to platelet release. This is associated with the stimulation of thrombopoietin generation by IL-6 and with a direct effect of this cytokine on megakaryocytes. IL-6 causes an increase in the ploidy of megakaryocytic nuclei and an increase in cytoplasm volume, which in consequence leads to the production of a large number of blood platelets [56].

The course of an inflammatory condition is also associated with increased percentage of large platelets, probably due to intracellular synthesis of procoagulatory and proinflammatory factors, degranulation of granules, and initiation of the platelet pool stored in the spleen [57]. Simultaneously, these cells rapidly migrate to the site of inflammation where they undergo activation and wear [58]. This seems to explain the drop in MPV in patients with ongoing inflammation [59].

### 3.1. MPV in Cardiovascular Diseases

It is currently suggested that changes in MPV can be considered and used as a prognostic factor in a number of inflammatory diseases. It has been shown that hyperreactivity of blood platelets markedly increases individual susceptibility of patients to acute cardiac incidents. A study conducted by Endler et al. [2] revealed that irrespective of how advanced coronary disease is, the increased platelet volume is associated with a higher risk of acute cardiac incidents. The presence of large and reactive platelets also increases the risk of thrombus formation after atherosclerotic plaque rupture [2, 60]. Potential effects of diabetes, smoking, and hypertension on MPV have also been considered in the study. The authors suggest that MPV ≥ 11.6 fl can also be an independent risk factor of heart infarct in patients with coronary disease and designate patients threatened with acute cardiac incidents. Also, Slavka et al. [3] observed that elevated MPV can be an independent high risk factor of death in patients after acute ischemic cardiac incident. They showed that standard treatment, i.e., aspirin, does not affect MPV, whereas clopidogrel significantly decreases the ADP-dependent MPV increase [3].

Huczek et al. [4] indicate that in patients with STEMI infarct treated with transdermal cardiac intervention, MPV is a potent and independent predictor/factor of increased number of restenosis after cardiac angioplasty and higher mortality rate. After the procedure, over 25% of patients have elevated platelet counts and MPV, which enhance the risk of cardiovascular incidents as compared to subjects with normal platelet count and size. This allows designation of a subgroup of patients with MPV ≥ 10.3 fl, in which mortality rate is considerably higher in a six-month period after the procedure [4]. In turn, Shah et al. [5] found no correlation between MPV and long-term survival of patients before transdermal coronary intervention. However, the increase in MPV after the procedure was associated with higher risk of death within a long time, assessed in a series of measurements performed to evaluate the dynamics of changes in MPV.

On the other hand, Gladwin and Martin [61] demonstrate that hypoxia increases the production as well as destruction of blood platelets, leading to MPV growth. A similar increase in platelet turnover caused by the action of prionflammatory cytokines can be observed in diabetic patients and smokers [62]. These observations can explain the fact that antiplatelet therapy reduces the risk of complications and in the future also cardiovascular incidents in patients undergoing heart revascularization procedure [55, 63]. Also, in patients with STEMI infarction, who showed elevated MPV, the application of abciximab decreased mortality [55].

### 3.2. MPV in Cerebrovascular Ischemia

High MPV values are also encountered in patients with acute cerebrovascular ischemia. Patients with high MPV were more at risk of acute stroke than those with normal MPV [7]. Butterworth and Bath [64] showed increased platelet volume 3 months after severe stroke. O'Malley et al. [8] demonstrated a statistically significant increase in MPV and a drop in platelet count in patients with acute cerebral ischemia as compared to the control group. Moreover, MPV did not correlate with a six-month survival of patients. Interestingly, D'Erasmo et al. [6] showed a significant increase in MPV and a decrease in PLT in patients after cerebral stroke. The authors reported higher mortality among patients with significantly increased MPV than in patients who survived. The authors suggest a possible use of MPV determinations as a prognostic marker in patients after stroke [6, 8].

### 3.3. MPV in Respiratory Diseases

Differences in platelet size have also been observed in respiratory diseases, accompanied by an inflammatory condition. In active tuberculosis, cellular body immunity is stimulated. Bacillus-activated macrophages and lymphocytes produce such cytokines as IL-6 or TNF-*α*, which affect maturation of megakaryocytes and platelet release. Therefore, reactive thrombocytosis is observed in tuberculosis, in which increased platelet count and size are associated with inflammation intensity [9, 10]. Contrary to the stable form, during disease exacerbation, the MPV is significantly reduced. The decrease in MPV can be related to the formation of microthrombi in tuberculous cavities, which are to inhibit the disease spread and are considered a defense reaction [27]. It has been suggested that MPV can be a negative marker of the acute phase of inflammation, and a decrease in MPV can be caused by increased platelet count and their accelerated wear.

In chronic sinusitis, as reported by Koç et al. [11], the platelet count is at the upper normal limit as compared to healthy subjects. Also, MPV is significantly higher in this group of patients. This may suggest the involvement of blood platelets in the development and course of the disease [11].

### 3.4. MPV in Other Diseases Accompanied by Inflammation

Chronic renal failure is frequently accompanied by reduced platelet aggregation and in consequence prolonged bleeding time. The application of therapy with recombinant erythropoietin (HuEPO) significantly improves hemostasis but increases the risk of thrombotic events [65]. Receptors for EPO are observed on the surface of megakaryocytes and their activation promotes thrombocytopoiesis. Sharpe et al. [12] showed a significant increase in MPV in patients with chronic renal failure treated with recombinant human erythropoietin. The authors suggest that HuEPO stimulation causes a release of young thrombocytes that are larger and undergo activation and aggregation more easily, leading to higher risk of blood clot formation [12]. The researchers excluded the effect of dialysis and aspirin administration on MPV, and even the increase in MPV was not EPO dose-dependent. MPV is considered a valuable marker to differentiate between patients and healthy subjects in Crohn's disease [13]. Zubcevic et al. [14] showed MPV to be a reliable marker of the disease activity, but its sensitivity is too low to differentiate moderate from severe form of the disease. On the other hand, the increased activity of ulcerative colitis is associated with MPV decrease [28]. However, not all studies seem to confirm the diagnostic usefulness of MPV in inflammatory diseases of the intestines. Kapsoritakis et al. [66] failed to observe significant changes in the level of MPV in patients with ulcerative colitis and in Crohn's disease. They also found no correlation between the disease activity and MPV.

In recent years, it is suggested that MPV can be linked to the activity of rheumatoid arthritis (RA); however, the data on this subject have still been controversial [67]. Yazici et al. [68] showed that MPV correlated with inflammatory markers (ESR and CRP) and disease activity (DAS-28 score) in RA patients. Also, Şahin et al. [69] in RA patients observed that MPV inversely significantly correlated with ESR and CRP and negatively correlated with DAS-28 score. The authors concluded that the lower MPV level surrogates active and/or chronic inflammatory state in the body [69]. Moreover, MPV can be used as a negative acute-phase biomarker in rheumatic diseases. The opposite results are presented by Moghimi et al. [70], as they showed that MPV value may not be able to predict disease activity in RA patients. The authors stating that the limitation of their examination may be the small group of patients [70]. According to Gasparyan et al. [15] in rheumatoid arthritis, low MPV values are associated with disease exacerbation and are likely caused by an increase in the wear of blood platelets at the site of inflammation. Increased MPV is observed due to administration of anti-inflammatory drugs that modify the course of the disease or biological drugs (anti-TNF alpha) [15].

The diagnostic usefulness of MPV was also assessed in the course of juvenile systemic lupus erythematous (SLE). Study results showed higher MPC levels in pediatric patients as compared to the control. El-Garf et al. [71] observed no correlation between MPV and disease advancement in children with SLE. The study group supervised by Yavuz and Ece [16] showed higher MPV levels in children with SLE, although contrary to other researchers, they reported a significant correlation between MPV and SLE advancements and even suggested possible application of MPV as an additional marker, apart from ds-DNA and C3.

Contrary to the results obtained in the group of children with juvenile SLE, adult patients with active disease had a statistically significant decrease in MPV values as compared to inactive SLE. Researchers suggest that this is due to the consumption of large active blood platelets during inflammation, although they also indicate that this is only a theory to be further confirmed by clinical research [29].

Altered platelet morphology, metabolism, and function were found also in diabetic patients [17, 72, 73], and higher platelet reactivity is the main factor responsible for increased risk and worse outcome in these patients [17, 18]. Elevated MPV was reported in patients with diabetes and impaired fasting glucose subjects as compared to nondiabetes individuals [17, 73, 74]. According to Tschöpe et al. [75], increased MPV and the number of glycoprotein receptors on platelets of diabetes patients indicate that the process of thrombopoiesis is altered in these patients and occurs at the beginning of diabetes. In addition, Papanas et al. [17] reported also positive correlation between elevated MPV and microangiopathic diabetes-related complication such as retinopathy and nephropathy. Interestingly, some authors showed that the percentage of glycated haemoglobin (HbA1_C_) did not have any influence on MPV [76]. On the other hand, studies of Demirtunc et al. [77] and Coban et al. [78] are in opposite to these results, as they revealed that HbA1_C_ may have impact on MPV. Kadić et al. [79] recommended the MPV value 9.55 fl as an indicator of poor glucoregulation.

## 4. MPV in Carcinoma

Recently, increasing attention has been paid to the assessment of MPV in cancer patients. Neoplastic transformation is associated with a chronic inflammatory process, which may also affect platelet parameters.

### 4.1. MPV in Carcinomas of the Gastrointestinal Tract

Hepatocellular carcinoma (HCC) is one of the most common primary cancers of the liver. The first symptoms of tumor growth are masked by chronic liver diseases (CLD), such as cirrhosis or viral infections [19]. Kurt et al. [19] suggest that in patients with chronic liver diseases, MPV can be a potential marker of liver cancer. The authors showed that MPV levels in patients with HCC were significantly higher as compared to patients with chronic hepatitis and in healthy subjects. Moreover, the level of MPV was significantly higher in patients with liver cirrhosis and hepatocellular carcinoma as compared to patients suffering only from liver cirrhosis [19]. Thrombocytopenia and a significantly increased MPV in these patients may result from decreased activity of thrombopoietin and bone marrow suppression associated with chronic HCV infection and antiviral therapy application [80]. Also, Cho et al. [81] aimed to assess the changes in the platelet count and MPV/PLT ratio in patients with hepatocellular carcinoma. The authors showed that both the MPV value and the MPV/PLT ratio were significantly higher in patients with carcinoma than in healthy patients. In the study group, the marker was the highest among women.

According to literature data, studies concerning colorectal cancer and MPV yield divergent results. A study conducted by Karagöz et al. [82] showed a higher platelet count in colorectal cancer patients as compared to healthy subjects and lack of differences in MPV between the study group and control. Moreover, no changes were noted in MPV between patients with and without colorectal cancer metastases. MPV was not found to correlate with cancer location and advancement [82]. On the other hand, research performed by Li et al. [20] demonstrated a significantly higher MPV level in colorectal cancer patients than in healthy subjects. Moreover, the researchers observed a significant decrease in the level of MPV after surgical removal of intestinal tumor and therefore they referred to MPV as to an independent prognostic factor of patients' survival after intestinal tumor resection [20]. Similar findings were reported by Tunce et al. [21] in patients with colorectal cancer prior to the application of bevacizumab-combined chemotherapy. They observed a significant increase in MPV in patients with metastases (mCRC) as compared to nonmetastatic colorectal cancer patients (non-mCRC), suggesting that these differences result from considerable enhancement of the inflammatory process and platelet activation in more advanced metastatic disease. At the same time, they failed to find any difference in the platelet count between the study groups of patients. They also showed that patients with decreased MPV before chemotherapy responded much better to the therapy applied, achieving longer remission [21]. Inanc et al. [30] examined the effect of chemotherapy (XELOX and XELOX-bevacizumab) on MPV levels in colorectal cancer patients. Prior to treatment, MPV findings were similar in the whole study group, although after three therapeutic cycles, the MPV values dropped in all patients. However, no differences were noted in MPV between patients undergoing various chemotherapies. Interestingly, patients receiving XELOX-bevacizumab showed an increase in platelet count and in the incidence of thrombotic events as compared to patients given XELOX alone. The authors indicate that changes in MPV could be due to the effect of chemotherapy on the formation of blood platelets and cyclic drug administration [30].

Gastric cancer is another gastrointestinal lesion accompanied by changes in the platelet parameters. It is characterized by early metastasis formation and high mortality [83]. Kilinçalp et al. [22] report that in patients with primary gastric cancer, MPV was considerably higher before surgery as compared to healthy subjects. The level of MPV did not correlate with the disease advancement. Following the surgery, the level of MPV underwent a significant reduction to the values comparable to control. Research conducted by Shen et al. [31] confirmed earlier reports on MPV in gastric cancer patients. The researchers also observed a high preoperative level of MPV in patients and its significant decrease when the treatment was applied. They additionally analyzed the patients' survival depending on the MPV value, showing that low MPV prior to therapy is a good prognostic factor of survival and recovery after gastric tumor resection [31]. The development of gastric cancer has been known to be closely associated with chronic inflammation accompanied by significantly elevated IL-6 concentration [84]. This cytokine via receptor binding on the surface of megakaryocyte progenitor cells causes their maturation and proliferation and in consequence enhances platelet release. The authors suggest that the increased MPV in gastric cancer patients can be a sequel of inflammatory condition and the accompanying elevated level of IL-6. Likewise, Matowicka-Karna et al. [23] observed an increase in IL-6 in gastric cancer patients. The authors demonstrated that MPV and PLT in patients in an early stage of cancer were similar to those found in healthy subjects and increased with the disease progression. However, after tumor resection, a further increase was noted in the platelet count and the percentage of large platelets was elevated (LPLT > 20 fl). In the authors' opinion, the occurrence of correlations between LPLT and MPV may confirm the relationship between changes in the morphological parameters of blood platelets and growing inflammation in cancer patients [23].

The analysis of study results in patients with neuroendocrine tumor of the pancreas shows that prior to surgery the level of MPV was statistically significantly lower as compared to patients suffering from pancreatic adenocarcinoma. Therefore, they emphasize that MPV can be a useful marker to differentiate between these two cancers [32]. In turn, a study conducted by Gong et al. [24] demonstrates that MPV was significantly higher in patients with pancreatic cancer prior to surgery as compared to those having benign lesions of the organ and healthy subjects. After surgery, the MPV value decreased significantly. The authors also assessed the PC/MPV value, i.e., platelet count to volume ratio, to find its high level before surgery and a significant decrease after the operation [24]. Moreover, the researchers demonstrate that the PC/MPV is an independent prognostic factor of patients' survival after pancreatic tumor removal. High value of the marker is a poor prognostic factor due to the release of growth and prothrombotic factors from platelets, which affect angiogenesis and progression of neoplastic disease [24]. Also, Yin et al. [25] indicated that elevated MPV (cut-off value of 8.7 fl) is associated with worse survival outcome in patients with pancreatic cancer with synchronous liver metastases.

Zhang et al. [85] assessed the diagnostic usefulness of the platelet count-mean platelet volume (COP-MPV) combination. The researchers searched for a prognostic factor in patients with squamous cell carcinoma of the esophagus, which could be determined in routine blood tests. Their study results demonstrate that simultaneous determination of platelet count and volume has a higher prognostic value both at an early stage and in advanced disease, especially in the assessment of lymph node involvement. The authors show that separate determination of platelet parameters has no such significance as COP-MPV, especially in the assessment of survival before surgical removal of esophageal tumor [85].

### 4.2. MPV in Other Cancers

Changes in MPV were observed in patients with papillary thyroid cancer [50]. An increase in MPV was found even in small neoplastic lesions and correlated with tumor growth. A positive correlation was noted between MPV and the level of thyroglobulin in these patients. The authors indicate that the increase in the parameter may also affect the risk of cardiovascular events and clot formation in the affected patients [26].

Some authors indicated that MPV was associated with renal cell carcinoma (RCC) [33, 86] which is the most common kidney malignancy. It was assessed whether the administration of antiangiogenic TKIs (sunitinib, sorafenib, or pazopanib) affects the MPV value in patients with renal cell carcinoma [86]. The results revealed elevated MPV levels after three months of treatment. The antiangiogenic therapy significantly prolongs survival without cancer relapse but at the same time increases the risk of thrombotic events in these patients. It has been known that high MPV values also correlate with thrombosis in cancer patients, which is most probably associated with by far greater number of fibrinogen receptors on large thrombocytes. Therefore, the authors suggest a potential beneficial effect of antiplatelet drug administration in RCC patients treated with antiangiogenic TKIs [33]. In turn, Yun et al. [33] demonstrate that MPV is reduced in RCC, which contradicts earlier findings. However, the researchers put forward a few theories suggesting a likely cause. They think that the inflammatory condition accompanying the carcinoma may lead to excessive platelet consumption and in consequence to MPV decrease, which has been confirmed lately [87]. The authors also indicate that a drop in MPV may result from blood platelet involvement in angiogenesis, migration, and invasion of cancer cells.

As revealed by literature analysis, an increase in MPV is observed in the majority of neoplastic diseases, although in some cancers, a decrease may be found. Inagaki et al. [34] in patients with non-small-cell lung cancer showed a significant reduction in MPV and MPV/PC ratio. The authors believe that the decrease in these parameters is caused by inverse nonlinear correlation between platelet count and their volume. This means that the enhanced release of blood platelets by megakaryocytes, stimulated by the action of the inflammatory cytokines IL-6 and IL-3, leads to a decrease in MPV. As we have already mentioned, some studies indicate that large-volume blood platelets are more reactive than the small ones and therefore undergo activation more rapidly, which leads to their faster consumption. A study by Kumagai et al. [35] confirmed a lower MPV level in patients with lung cancer as compared to healthy subjects in the control group. Moreover, the authors assessed the prognostic value of MPV to show that low preoperative MPV level is an independent unfavorable prognostic factor in patients after total cancer resection [35]. Reduced MPV values were also observed in patients with cancer of the uterine cervix. The scientists reached the conclusions that low MPV values are independently related to the presence of cancer in the affected women and that the mechanisms leading to MPV reduction are unknown. However, they put forward a few likely theories. One of them involves bone marrow dysregulation associated with cancer development and first of all with the production of considerable amounts of IL-6. On the other hand, they point to platelet engagement in the formation of cancer metastases as a potential cause. However, these are only suggestions that require further molecular research [36].

## 5. Factors Influencing PLT and MPV Values

Some researchers indicate that MPV should always be assessed together with platelet count as there is a nonlinear inverse relationship between PLT and MPV [88]. However, also other factors like age, gender, race and ethnicity, lifestyle (including diet), and genetic factors may strongly influence MPV and PLT [89–93]. A high heritability of 84% and of 75% for PLT and MPV, respectively, has been showed due to genetic variations [93].

Vasudeva and Munshi [94] in their review strongly highlight the role of genetic variants in platelet reactivity response at the site of injury of the vessel wall. Interindividual genetic variations of platelet reactivity (in terms of PLT and MPV) significantly modulate the course of thrombotic events. Therefore, an identification of genetic effects on PLT and MPV may become a potential therapeutic target [94].

Genetic variants are classified into (1) rare mutations (variants) with large effect, (2) common polymorphisms with small effects, and (3) polymorphisms with major effects. According to Kunicki et al. [92], polymorphisms with small effects are mainly responsible for hyperactive platelet phenotype. By means of high-throughput techniques, e.g., GWAS (genome-wide association studies), WES (whole exome sequencing), WGS (whole genome sequencing), PheWAS (phenome-wide association studies), large-scale Exomechip genotyping array, and multiomic analysis, different genetic variants influencing PLT and MPV so far have been recognized [90, 94]. For example, GWAS studies have indicated 68 distinct loci associated with PLT and MPV [90]. Candidate genes related with platelet reactivity response include genes associated with megakaryopoiesis, megakaryocyte/platelet adhesion, platelet formation, regulation of the cell cycle, or platelet surface receptors [91, 92]. However, the heritability of these genetic variations still remains not fully understood [90].

Yazici et al. [89] investigated the role of lifestyle modification: weight loss recommended for those participants with BMI ≥ 25, dietary sodium (Na^+^) intake < 100 mEq/day, increased physical activity, limited alcohol intake, and Dietary Approaches to Stop Hypertension (DASH) on MPV value in prehypertension (PHT) individuals. The abovementioned lifestyle changes were required from studied individuals for 20 weeks. Authors showed that recommended approaches significantly reduced MPV in PHT subjects, which suggest that the style of living may play a role in decreasing platelet activation and may become an aspect of therapy in these patients. However, their study cannot be extrapolated to all PHT individuals, as Yazici et al. excluded cardiovascular disease patients and study was limited to only to young and overweight Caucasian adults [89].

Hou et al. [95] on a cohort of *n* = 27,009 Chinese individuals analyzed the association of MPV with adiposity. They showed that in females MPV was negatively related to waist circumstance (WC) and waist-to-height ratio (WHtR) [95]. Another large cohort study (*n* = 10,619 adult population of the United States), performed by Ijaz et al. [96], presented that serum L-thyroxine (T4) is associated with PLT, while there was no relationship between hypophysis-thyroid axis hormones and MPV. Also, Abudesimu et al. [97] presented interesting findings, who analyzed platelet parameters in the group of patients with hypertension subtypes among the Han, Uygur, and Kazakh ethnic groups and their associated risk factors in Xinjiang, northwestern China (*n* = 9816 adult participants). Authors found that risk factors for MPV included Uygur ethnicity, smoking, overweight, obesity, isolated systolic hypertension, isolated diastolic hypertension, diabetes, and high triglycerides [97]. Based on the obtained results, Abudesimu et al. highlight that more attention should be paid to factors that may affect platelet parameters, including PLT and MPV, as they may have an effect on antiplatelet treatment. Also, Cho et al. [98] found that smoking may have an effect on the MPV value, as smokers had higher MPV values compared to nonsmokers; however, significant differences were found only for females, which indicates that gender aspect in terms of platelet reactivity is important.

Data concerning the MPV value depending on gender is conflicting [3, 54, 99–104]. Some authors identified higher MPV values in women [54, 99, 100], while others in men [103]. We also can find studies reporting no significant differences for MPV value between women and men [3, 88, 102, 104, 105].

According to some authors, also the hormonal profile may affect MPV value; however, also in this aspect, obtained results are contraindicatory [106–109]. Butkiewicz et al. [106] tried to analyze whether the lack of estrogens influences platelet morphological parameters, including MPV. They showed that PLT in healthy postmenopausal women without estrogen replacement therapy was lower than that in healthy women before menopause; nonetheless, for MPV, authors did not present any significant differences between the groups analyzed [106]. Bulur et al. [108] and Saleh et al. [109] reported no evidence for altered platelet function and MPV in women taking oral contraceptives. On the other hand, Panova-Noeva et al. [107] demonstrated that oral contraceptive intake by females was strongly related with higher MPV values.

The use of certain antithrombotic drugs may also affect MPV. It has been demonstrated that aspirin does not affect MPV, but no data are available on the potential effect of other antiplatelet drugs on changes in this parameter [110].

**Figure 1:**
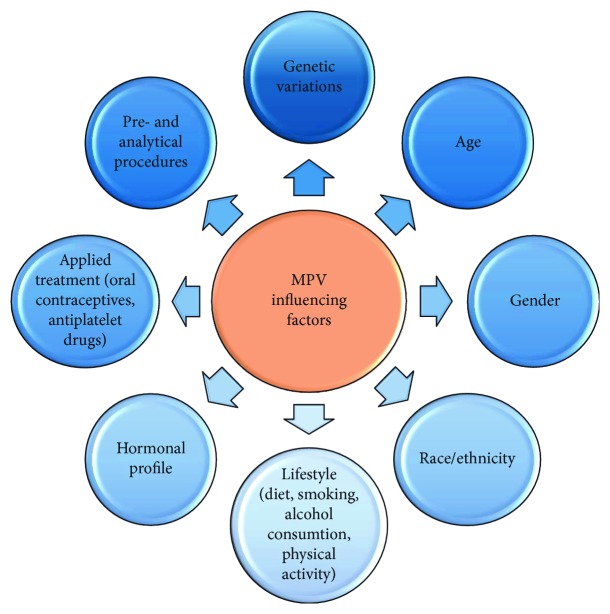


Available literature presented in this subsection demonstrates that the aspect of multiple determinants influencing PLT and MPV in physiology and during disease state is not so easy to understand, as different factors may affect platelet reactivity (Figure 1). In the future, much effort also should be put into linking the role of genetic variations regulating platelet traits during different pathology conditions.

## 6. Preanalytical and Analytical Factors Influencing MPV

Nowadays, MPV evaluation is widely available in clinical laboratories as it is routinely measured within complete blood count (CBC) test. Although this parameter has been evaluated for many decades, the intercenter comparison of MPV still has been suffering from lack of standardization of the preanalytical as well as analytical procedures (Figure 1), different hematological analyzers and methods used, and the lack of universal external calibration [111–114].

Among the preanalytical factors that may influence MPV value, we can distinguish (1) the venipuncture method (with or without stasis), (2) the appropriate filling of the tube with blood, (3) the accuracy of sample mixing, (4) the anticoagulant used for blood collection (centrifugation of the blood collected into citrate anticoagulant may lead to platelet activation resulting in the presence of more active large platelets; the use of EDTA as an anticoagulant may cause platelet swell, which moreover is time-dependent), (5) the type of sample (change in MPV value is smaller in platelet rich plasma compared to the whole blood). Also, the temperature of the blood being analyzed affects MPV value: cooling the sample to room or lower temperature increases MPV; on the other hand, rewarming sample to body temperature of 37°C restores platelets to their initial size [113, 115–120].

Diversity of methods used for platelet morphology evaluation is another factor responsible for MPV value differences between laboratories [121]. According to Jagroop and Mikhailidis [122], more attention should be given to the interpretation of the MVP value depending on the method used for platelet size evaluation [122]. Hematological analyzers generally are based on the impedance method or on the optical method with the use of laser light scatter [114]. Some hematological analyzers may also use both or even more methods, e.g., impedance, optical, and immunological methods, however mainly for PLT measurement, rather not for MPV estimation [115].

Imprecision of MPV evaluation by an impedance analyzer results from the presence in the small/fragmented red blood cells (RBC) or blasts (cell debris), as all small particles, which size falls within a specific size range, are recognized as platelets. On the contrary, a so-called giant platelet can be falsely interpreted as RBC [123, 124]. This analytical bias may lead to falsely increased platelet count, which in turn causes inability to give an appropriate MPV value, or may result with giving a false MPV value, based only on the platelet population being measured [125]. In optical analyzers, platelets are recognized based on their volume (forward scatter) and density (side scatter) [121]. Using the optical method may result in a situation in which microcytes are counted as large platelets [125].

In the available literature, there is also no consensus regarding “large platelet” definition. For example, the term “macroplatelets” refers to large platelets measured by means of impedance or an optical method and also corresponds to platelet size lower than RBC on May-Grünwald Giemsa (MGG) staining blood smear. On the contrary “giant platelets” refers to large platelets which can be analyzed with the use of an optical but not impedance method and corresponds to platelet size higher or equivalent to RBC on MGG blood smear [125]. To standardize platelet size definition, Latger-Cannard et al. [125] suggest to refer MPV to the platelet volume distribution curve together with platelet morphological analysis. However, still the reference method used for the evaluation of platelet size is a microscopic examination of the MGG blood smear with a parallel evaluation of the platelet morphology by an experienced cytologist [125].

Another issue that should be taken into account is the aspect of the MPV threshold value, as the available literature presents different cut-off points depending on the method and hematological analyzer used [125–127]. Pathepchotiwong et al. [126], using the Beckman Coulter STKS analyzer (impedance method), in the healthy population received an average MPV value of 7.90 fl (5.60-10.90 fl). Noris et al. [127] also evaluated the mean value of MPV in a healthy population, but using two different analyzers, and received different mean values depending on the analyzer. Authors obtained the average MPV value 8.20 fl (7.80-8.70 fl) using the ADVIA 2120 analyzer (optical method) and the value 10.50 fl (10.20-11.20 fl) measured with the XE-2100 analyzer (impedance and optical methods based on fluorescence) [127]. Similarly, Latger-Cannard et al. [125] found that the average MPV value is about 20-25% lower when measured with the ADVIA 2120 analyzer compared to the XE-2100 analyzer. This clearly indicates the need to establish individual reference values for MPV by laboratories. Noris et al. [121] highlight that to indicate appropriate MPV normal range, each laboratory should enroll adequate number of individuals with respect to gender, age, and even ethnicity [121].

Finally, we do not have a universal external calibrator for MPV [125], and moreover, the accuracy of MPV evaluation might be inadequate because of the control samples, which quality may worsen when getting close to the expiry date [121].

Undoubtedly, to gain more benefits in terms of clinical purpose from MPV evaluation by means of hematological analyzer, the clinical laboratories should strive for standardization of both the preanalytical and analytical phases. Only then the MPV value may have a chance to develop a broader clinical application.

## 7. Conclusions

The study of MPV, which is a routine test performed during blood morphology, can provide important information on the course and prognosis in many inflammatory conditions. However, to gain more benefits in terms of a clinical purpose from MPV evaluation, the clinical laboratories should strive for standardization of both the preanalytical and analytical phases.

Increased MPV was observed in cardiovascular diseases, cerebral stroke, respiratory diseases, chronic renal failure, intestine diseases, rheumatoid diseases, diabetes, and various cancers. Decreased MPV was noted in tuberculosis during disease exacerbation, ulcerative colitis, SLE in adult, and different neoplastic diseases. Therefore, from the clinical point of view, it would be interesting to establish MPV cut-off value indicating the intensity of inflammatory process, presence of the disease, increased risk of disease development, increased risk of thrombotic complications, increased risk of death, and patient's response on applied treatment. Nevertheless, this aspect of MPV evaluation allowing its use in clinical practice is limited and requires further studies.

## Figures and Tables

**Table 1:** Increased MPV in various diseases [2–26].

*↑MPV in cardiovascular diseases*	
Endler et al. 2002 [2]	Associated with a higher risk of acute cardiac incidents
Slavka et al. 2011 [3]	Independent high risk factor of death in patients after acute ischemic cardiac incident
Huczek et al. 2005 [4]	Related to the increased mortality rate in a six-month-period after the transdermal cardiac intervention
Shah et al. 2013 [5]	Associated with higher risk of death within a long time after the transdermal cardiac intervention
↑*MPV in cerebral stroke*	
D'Erasmo et al. 1990 [6]	Increased MPV after stroke was related to higher mortality
Greisenegger et al. 2004 [7]	Associated with a risk of acute stroke
O'Malley et al. 1995 [8]	MPV as a prognostic marker in patients after stroke characteristic for acute and nonacute phases of cerebral ischemia
↑*MPV in respiratory diseases*	
Feng et al. 2011 [9] Unsal et al. 2005 [10]	In tuberculosis associated with intensity of inflammation
Koç et al. 2011 [11]	Associated with the development and course of the chronic sinusitis
↑*MPV in chronic renal failure*	
Sharpe et al. 1994 [12]	In patients treated with recombinant erythropoietin (HuEPO) leading to higher risk of blood clot formation
↑*MPV in intestine diseases*	
Liu et al. 2012 [13]	Differentiate patients with Crohn's disease from healthy subjects
Zubcevic et al. 2010 [14]	Marker of the Crohn's disease activity
↑*MPV in rheumatoid diseases*	
Gasparyan et al. 2010 [15]	In rheumatoid arthritis, increased MPV is observed due to administration of anti-inflammatory drugs
Yavuz and Ece 2014 [16]	Correlated with the advancement of juvenile SLE
↑*MPV in diabetes*	
Papanas et al. 2004 [17]	Associated with retinopathy and nephropathy
Rollini et al. 2013 [18]	Responsible for increased risk of diabetes and worse outcome
↑*MPV in carcinomas*	
Kurt et al. 2012 [19]	Potential marker of liver cancer in patients with chronic liver diseases
Li et al. 2017 [20]	In colorectal cancer patients compared to healthy subjects
Tunce et al. 2014 [21]	In colorectal cancer patients with metastases compared to nonmetastatic colorectal cancer patients
Kılınçalp et al. 2014 [22]	In primary gastric cancer patients preoperative level considerably higher compared to healthy subjects
Matowicka-Karna et al. 2013 [23]	In preoperative gastric cancer associated with the disease progression
Gong et al. 2016 [24]	In preoperative pancreatic cancer patients compared to those having benign lesions of the organ and healthy subjects
Yin et al. 2018 [25]	Associated with worse survival outcome in patients with pancreatic cancer with synchronous liver metastases
Carlioglu et al. 2014 [26]	Observed in papillary thyroid cancer patients, correlated with tumor growth

**Table 2:** Decreased MPV in various diseases [15, 20, 22, 24, 27–36].

↓*MPV in tuberculosis*	
Gunluoglu et al. 2014 [27]	During disease exacerbation can be related to the formation of microthrombi in tuberculous cavities
↓*MPV in ulcerative colitis*	
Yuksel et al. 2009 [28]	Associated with increased activity of ulcerative colitis
↓*MPV in rheumatoid diseases*	
Gasparyan et al. 2010 [15]	In rheumatoid arthritis associated with disease exacerbation
Delgado-Garcia et al. 2016 [29]	In adult patients can be related to active SLE
↓*MPV in carcinomas*	
Li et al. 2017 [20]	An independent prognostic factor of patients' survival after intestinal tumor resection
Inanc et al. 2014 [30]	In colorectal cancer patients effect of chemotherapy (XELOX and XELOX-bevacizumab)
Shen et al. 2016 [31]	Prior to therapy is a good prognostic factor of survival and recovery after gastric tumor resection
Kılınçalp et al. 2014 [22]	In primary gastric cancer patients significant reduction after surgery
Karaman et al. 2011 [32]	A useful marker in differentiation patients with neuroendocrine tumor of the pancreas from pancreatic adenocarcinoma
Gong et al. 2016 [24]	In pancreatic cancer patients significant reduction after surgery
Yun et al. 2017 [33]	In renal cell carcinoma patients may be a result of (1) inflammation which probably lead to excessive platelet consumption and/or (2) platelet involvement in angiogenesis, migration, and invasion of cancer cells
Inagaki et al. 2014 [34]	In patients with non-small-cell lung cancer resulted from inverse nonlinear correlation between platelet count and their volume
Kumagai et al. 2015 [35]	In lung cancer, low preoperative level is an independent unfavorable prognostic factor in patients after total cancer resection
Shen et al. 2017 [36]	Independently related to the presence of cancer of the uterine cervix
